# Frequency and Prognosis of Pulmonary Metastases in Newly Diagnosed Gastric Cancer

**DOI:** 10.3389/fonc.2019.00671

**Published:** 2019-07-30

**Authors:** Zepang Sun, Hao Liu, Jiang Yu, Weicai Huang, Zhen Han, Tian Lin, Hao Chen, Mingli Zhao, Yanfeng Hu, Yuming Jiang, Guoxin Li

**Affiliations:** Department of General Surgery, Nanfang Hospital, Southern Medical University, Guangzhou, China

**Keywords:** gastric cancer, pulmonary metastases, frequency, prognosis, SEER

## Abstract

**Purpose:** The purpose of this study was to analyze the frequency and prognosis of pulmonary metastases in newly diagnosed gastric cancer using population-based data from SEER.

**Methods:** Patients with gastric cancer and pulmonary metastases (GCPM) at the time of diagnosis in advanced gastric cancer were identified using the Surveillance, Epidemiology and End Result (SEER) database of the National Cancer Institute from 2010 to 2014. Multivariable logistic regression was performed to identify predictors of the presence of GCPM at diagnosis. Receiver operator characteristics analysis was performed to significant predictors on multivariable logistic regression and was then assessed with Delong's test. Multivariable Cox regression was developed to identify factors associated with all-cause mortality and gastric cancer-specific mortality. Survival curves were obtained according to the Kaplan-Meier method and compared using the log-rank test.

**Results:** We identified 1,104 patients with gastric cancer and pulmonary metastases at the time of diagnosis, representing 6.02% of the entire cohort and 15.19% of the subset with metastatic disease to any distant site. Among the entire cohort, multivariable logistic regression identified six factors (younger, upper 1/3 of stomach, intestinal-type, T4 staging, N1 staging, and presence of more extrapulmonary metastases to liver, bone, and brain) as positive predictors of the presence of pulmonary metastases at diagnosis. The value of AUC for the multivariable logistic regression model was 0.775. Median survival among the entire cohort with GCPM was 3.0 months (interquartile range: 1.0–9.0 mo). Multivariable Cox model in SEER cohort confirmed five factors (diagnosis at previous period, black race, adverse pathology grade, absence of chemotherapy, and presence of more extrapulmonary metastases to liver, bone, and brain) as negative predictors for overall survival.

**Conclusions:** The findings of this study provided population-based estimates of the frequency and prognosis for GCPM at time of diagnosis. The multivariable logistic regression model had an acceptable performance to predict the presence of PM. These findings may provide preventive guidelines for the screening and treatment of PM in GC patients. Patients with high risk factors should be paid more attention before and after diagnosis.

## Introduction

Gastric Cancer (GC) was the fourth most common malignant tumor in the world and the fifteenth in the United States (1, 2). Although the reported incidence and mortality rates had steadily decreased over the last decade, there was still an estimated 26 240 new GC patients and 10 800 deaths in United States in 2018 (2). Furthermore, about 40% of patients were presented with evidence of distant metastases (3–5). The most common site of distant metastases was the peritoneum, followed by the liver, lung, and bone (5). Pulmonary metastases (PM) were really rare discovery, which had been reported in only 0.5–16% of the GC patients with distant metastases in clinical practice (6–8), but 22–52% of patients at postmortem examination (9, 10). However, all these patients were unselected, including synchronous and asynchronous metastatic patients. PM was associated with poor survival in patients with advanced gastric cancer. The 5-year survival of gastric cancer and pulmonary metastases (GCPM) was only 2–4% (6, 7, 11). And the median survival time was 4 months for both newly diagnosed PM and those asynchronous patients (6, 7, 12).

An early detection of pulmonary metastases was necessary to alter patient management and result in significant cost savings and medical resources savings by reducing unnecessary surgery or other treatments. Chest CT was not recommended routine assessment in current gastric cancer screening guidelines. However, multiple studies revealed that CT was more superior in identifying some metastatic nodules than plain chest radiography and conventional liner tomography (CLT) (13–15). And the conventional chest radiograph was always adopted at the initial screening examination in clinical practice, which may lead to missed diagnosis. Thus, a population-based study including a large sample was particularly important to determine which patients need to receive further examination.

There were only limited data regarding pulmonary metastases from gastric cancer at present, and the majority of objects included in these researches were asynchronous metastatic patients (6–8, 11, 16, 17). The study in newly diagnosed gastric cancer with pulmonary metastases was lacking, so the proportion, predictive factors, prognostic factors, and optimal strategy for these patients were unknown. Therefore, a study based on population level about GCPM to describe epidemiologic characteristics and prognosis was urgently needed.

The purpose of this study was to use data from the Surveillance, Epidemiology and End Results (SEER) database between 2010 and 2014 to survey the incidence proportion and predictive factors of pulmonary metastases at the time of cancer diagnosis among patients with gastric cancer on a population-based level. We also wanted to characterize prognostic factors on the survival of patients at diagnosis of gastric cancer with pulmonary metastases.

## Materials and Methods

### Study Population

Data was obtained from the SEER database, which was the largest publicly available cancer dataset and collected cancer data from 18 population-based cancer registries covering about 28 percent of the United States population (18). This database included information about cancer incidence as well as demographic information: age, gender, race, year at diagnosis, tumor staging, tumor size, treatment, marital status, insurance, education, family income, and so on. We used the SEERStat software version 8.3.4 published by SEER to identify eligible patients in this study, which we could get from the official network (https://seer.cancer.gov/). The SEERStat provided patients information up to 2014 based on the November 2016 submission, and it started to release metastatic information related to pulmonary metastases from 2010. Thus, we can get information about GCPM between 1 January 2010 and 31 December 2014 from SEERStat. Besides, pulmonary metastases included only the lung, but not pleura or pleural fluid in the SEER database.

**Figure 1:**
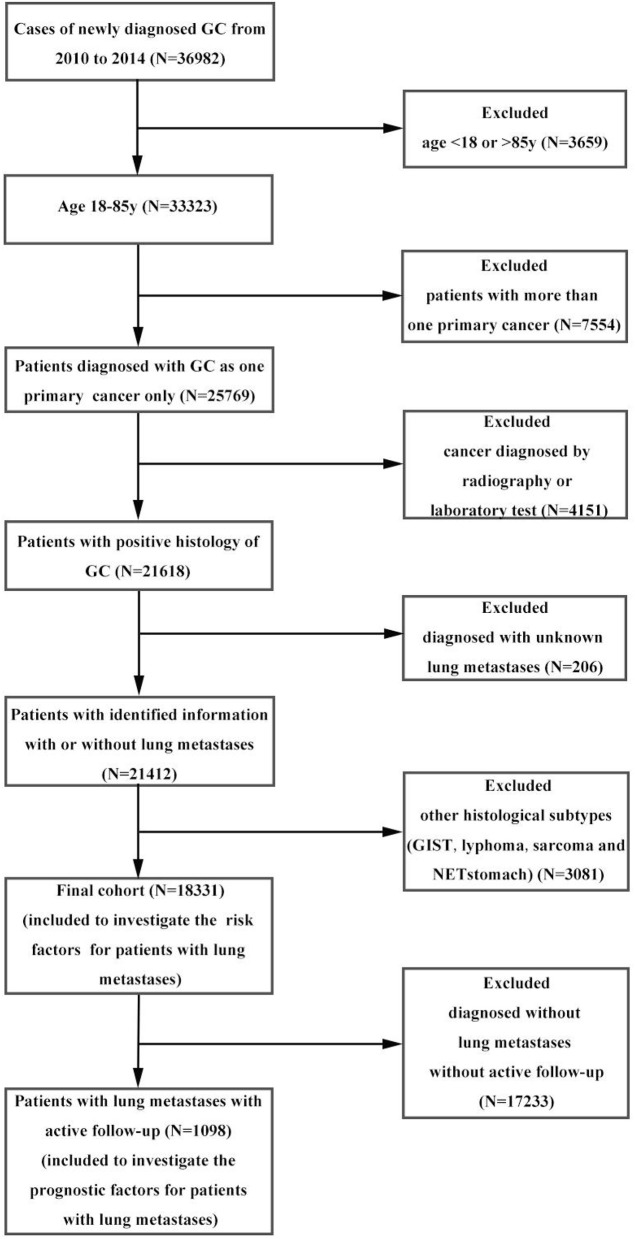
Data extraction flowchart from the SEER database.

Within the SEER database, we identified 36,982 patients with gastric cancer from 2010 to 2014. Patients with other cancers, <18 or more than 85 years old, with other pathological types were excluded from the analysis, leaving 18,331 patients in the final cohort for frequency analysis. Of these, 7268 patients were diagnosed with metastases to any distant site and 1,104 patients were diagnosed as GCPM. We subsequently removed patients with an unknown follow-up, leaving 1,098 patients eligible for survival analyses. The percentage of distant metastases to any site was 39.65% and pulmonary metastases were 6.02%. Data extraction flowchart was showed in Figure 1. The inclusion criteria were as follows: age more than 18 years old and <85 years old at time of diagnosis; gastric cancer as the only one primary cancer; with identified pulmonary metastases; confirmation of diagnosis based on pathology of a specimen, rather than based on radiography or laboratory; with active follow-up. And we excluded those patients conformed to any one of the following standards: age <18 years old or more than 85 years old at the time of diagnosis; with more than one primary cancer; unknown pulmonary metastases; cancer diagnosed by radiography or laboratory; pathological type confirmed to be NET stomach, sarcoma, GIST or lymphoma; without active follow-up. 12/31/2014 was the cut-off date in this study. More details can get from SEERStat software version 8.3.4 and SEER manual 2016. The end point of this study was OS. The OS was defined from the date of diagnosis to the date of all-cause death or cancer-specific death, and patients survived to the latest follow-up identified as censoring. Toward the last follow up, there were 925 deaths and 173 censoring among patients with GCPM.

### Statistical Analysis

Descriptive statistics was used to calculate the absolute number and frequency among patients with PM at the time of cancer diagnosis. Frequency was defined as the percentage of gastric cancer patients diagnosed with PM among the entire study cohort and the patients with metastatic disease to any distant site. All data were stratified by year at diagnosis, age, gender, race, original, primary site, pathology grade, Lauren classification, T staging, N staging, tumor size, treatment, number of extrapulmonary metastatic sites and other sociodemographic information, such as: marital status, residence type, insurance situation, bachelor education, median household income, and smoking status. Residence type, education level, median household income, and smoking status were defined by the county attributes from the US Census 2010–2014 American Community Survey 5-year data files, which we could get from the SEER^*^Stat software.

Chi-square or Fisher's test was developed for clinical characteristics of GCPM patients at the exclusion of those with unknown information. Multivariable logistic regression was used to determine predictors of the presence of pulmonary metastases at diagnosis. And only variables which demonstrated significance on both the Chi-square test and the univariate logistic regression can enter into the multivariable logistic regression. This was a population-based study, so we focused more on the entire cohorts (GC) but not subcohort (GC with metastatic disease to any distant site). Survival estimates were obtained according to the Kaplan-Meier method and compared using the log-rank test. Variables that reached significance with *P* < 0.05 were entered into the multivariable analyses using the Cox regression model to identify covariates associated with increased all-cause mortality. Besides, we used Fine and Gray's competing risk regression to assess gastric cancer-specific mortality (19). Binary-dependent receiver operator characteristics (ROC) analysis for different variables to predict the presence of PM was developed. And Delong's test was conducted to further expound the performance of multivariable logistic regression model.

All statistical analyses were performed using SPSS statistical software (version 18.0). The competing risks analysis was performed using the cmprsk package (version 2.2-7) and ROC was developed using the pROC package (version 3.2-5) in R (version 3.4.4; R Foundation). Delong's test was performed using Medcalc software. Statistical significance was set at two-sided (*P* < 0.05).

## Results

### Frequency Analysis

A total of 18,331 patients in the U.S. were diagnosed with gastric cancer between 2010 and 2014, including 1,104 patients diagnosed with GCPM whose median age was 66 years old, consisted of 773 men (70.02%) and 331 women (29.98%). Their demographic and clinical characteristics were shown in Table 1. On Chi-square or Fisher's test, a significant difference was found in age, gender, race, primary site, Lauren classification, T staging, N staging, tumor size, number of extrapulmonary metastatic sites, radiotherapy, surgery, insurance situation and median household income. The proportion of younger patients (age<60 years) (40.58 vs. 36.94% *P* < 0.001), male (70.02 vs. 65.08% *P* < 0.001) and white race (74.61 vs. 69.63% *P* < 0.001) in PM group was higher compared with the no-PM group. Furthermore, presence with more upper 1/3 of stomach (67.94 vs. 49.34% *P* < 0.001), extrapulmonary metastases (63.66 vs. 17.50% *P* < 0.001) and intestinal-type tumor (72.64 vs. 64.29% *P* < 0.001) could been seen in the PM group. Besides, T4 staging (35.73 vs. 24.93% *P* < 0.001), N1 staging (49.61 vs. 30.24% *P* < 0.001) and larger tumor (>2 cm) (85.89 vs. 79.25% *P* < 0.001) were significantly associated with PM. The sociodemographic information, like insurance situation and median household income, had little value in this study. However, the no-PM group presented with higher percentage of radiotherapy (30.15 vs. 20.11% *P* < 0.001) and surgery (48.77 vs. 4.62% *P* < 0.001). Additionally, only 51 patients received gastrectomy and 7 patients received gastrectomy plus metastectomy among the cohort with GCPM. Rate of chemotherapy showed no significant difference between PM group and no-PM group. More detail information can be found in Table S1.

**Table 1:** Clinical characteristics of patients with gastric cancer with identified pulmonary metastases at diagnosis.

**Variable**	**Patients, no**.			**Proportion of pulmonary metastases, %**		**Survival among patients with pulmonary metastases, median (IQR), mo**
	**With gastric cancer (*n* = 18,331)**	**With metastatic disease (*n* = 7,268)**	**With pulmonary metastases (*n* = 1,104)**	**Among entire cohort**	**Among subset with metastatic disease**	
**YEAR AT DIAGNOSIS**						
2010	3,492	1,409	221	6.33	15.68	2.0 (1.0–6.0)
2011	3,502	1,352	195	5.57	14.42	4.0 (1.0–9.0)
2012	3,751	1,469	218	5.81	14.84	3.0 (1.0–7.0)
2013	3,739	1,476	229	6.12	15.51	3.0 (1.0–11.0)
2014	3,847	1,562	241	6.26	15.43	4.0 (1.0–NA)
**AGE AT DIAGNOSIS, YEARS**						
18–40	773	451	63	8.15	13.97	3.0 (1.0–7.0)
41–60	6,039	2,763	385	6.38	13.93	4.0 (1.0–10.0)
61–80	9,682	3,512	581	6.00	16.54	3.0 (1.0–8.0)
80+	1,837	542	75	4.08	13.84	2.0 (0.0–7.0)
**RACE**						
White	12,735	5,182	820	6.44	15.82	4.0 (1.0–10.0)
Black	2,426	997	139	5.73	13.94	2.0 (1.0–7.0)
Others^a^	3,049	1,052	140	4.59	13.31	2.0 (1.0–7.0)
Unknown	121	37	5	4.13	13.51	14.0 (1–NA)
**GENDER**						
Male	11,984	4,743	773	6.45	16.30	3.0 (1.0–9.0)
Female	6,347	2,525	331	5.22	13.11	3.0 (1.0–8.0)
**ORIGINAL**						
Hispanic	3,862	1,690	216	5.59	12.78	3.0 (1.0–10.0)
Non-Hispanic	14,469	5,578	888	6.14	15.92	3.0 (1.0–9.0)
**PRIMARY SITE**						
Upper 1/3	7,027	2,681	553	7.87	20.63	4.0 (1.0–11.0)
Middle 1/3	1,676	694	70	4.18	10.09	3.0 (1.0–8.0)
Lower 1/3	3,808	1,186	119	3.13	10.03	3.0 (1.0–8.0)
Overlapping lesion	1,424	674	72	5.06	10.68	2.0 (1.0–5.0)
Unknown	4,396	2,033	290	6.60	14.26	2.0 (1.0–7.0)
**PATHOLOGY GRADE**						
I–II	4,742	1,397	269	5.67	19.26	6.0 (1.0–12.0)
III–IV	10,623	4,256	576	5.42	13.53	3.0 (1.0–7.0)
Unknown	2,966	1,615	259	8.73	16.04	2.0 (1.0–7.0)
**LAUREN CLASSIFICATION**						
Intestinal-type	11,878	4,621	802	6.75	17.36	3.0 (1.0–9.0)
Diffuse-type	5,552	2,241	232	4.18	10.35	3.0 (1.0–8.0)
Others^b^	901	406	70	7.77	17.24	3.0 (0.0–5.0)
**TUMOR STAGING^c^**						
I	3,315	0	0	NA	NA	NA
II	2,359	0	0	NA	NA	NA
III	4,351	0	0	NA	NA	NA
IV	7,268	7,268	1,104	15.19	15.19	3.0 (1.0–9.0)
Unknown	1,038	0	0	NA	NA	NA
**T STAGING^c^**						
T1	4,373	1,302	222	5.08	17.05	3.0 (1.0–8.0)
T2	1,650	333	35	2.12	10.51	3.0 (1.0–9.0)
T3	4,777	1,062	128	2.68	12.05	5.0 (2.0–11.0)
T4	3,672	1,642	214	5.83	13.03	2.0 (1.0–7.0)
Unknown	3,859	2,929	505	13.09	17.24	2.0 (0.0–9.0)
**N STAGING^c^**						
N0	7,775	2,548	362	4.66	14.21	3.0 (1.0–10.0)
N1	5,210	2572	442	8.48	17.19	4.0 (1.0–9.0)
N2	1,780	417	38	2.13	9.11	4.0 (1.0–10.0)
N3	1,893	496	49	2.59	9.88	3.0 (1.0–11.0)
Unknown	1,673	1,235	213	12.73	17.25	2.0 (1.0–6.0)
**M STAGING^c^**						
M0	11,063	0	0	0.00	0.00	NA
M1	7,268	7,268	1,104	15.19	15.19	3.0 (1.0–9.0)
**SURGERY^d^**						
Yes	8,453	883	51	0.60	5.78	4.0 (2.0–14.0)
No	9,878	6,385	1,053	10.66	16.49	3.0 (1.0–9.0)
**RADIOTHERAPY**						
Yes	5,416	1,251	222	4.10	17.75	5.0 (2.0–11.0)
No	12,915	6,017	882	6.83	14.66	2.0 (1.0–8.0)
**CHEMOTHERAPY**						
Yes	10,495	4,391	631	6.01	14.37	6.0 (3.0–13.0)
No	7,836	2,877	473	6.04	16.44	1.0 (0.0–2.0)
**TUMOR SIZE, CM**						
0–2	2,186	322	59	2.70	18.32	2.0 (0.0–9.0)
2–5	4,741	1,278	198	4.18	15.49	5.0 (1.0–12.0)
5+	3,742	1,226	161	4.30	13.13	3.0 (1.0–8.0)
Unknown	7,662	4,442	686	8.95	15.44	3.0 (1.0–8.0)
**EXTRAPULMONARY METASTATIC SITES TO LIVER, BONE, AND BRAIN, NO**.						
0	14,442	3,422	379	2.62	11.08	4.0 (1.0–13.0)
1	3,290	3,290	526	15.99	15.99	3.0 (1.0–8.0)
2	340	340	128	37.65	37.65	2.0 (1.0–8.0)
3	18	18	10	55.56	55.56	3.0 (1.0–6.0)
Unknown	241	198	61	25.31	30.81	1.0 (0.0–4.0)
**INSURANCE SITUATION**						
Yes	17,022	6,661	1,007	5.92	15.12	3.0 (1.0–9.0)
No	922	489	73	7.92	14.93	2.0 (1.0–7.0)
Unknown	387	118	24	6.20	20.34	2.0 (0.0–16.0)
**MARITAL STATUS**						
Married	10,618	4,194	627	5.91	14.95	4.0 (1.0–10.0)
Unmarried^e^	6,789	2,763	441	6.50	15.96	2.0 (1.0–7.0)
Unknown	924	311	36	3.90	11.58	3.0 (0.0–18.0)
**RESIDENCE TYPE**						
Rural	467	192	27	5.78	14.06	2.0 (1.0–6.0)
Urban	17,864	7,076	1,077	6.03	15.22	3.0 (1.0–9.0)
**BACHELOR EDUCATION (PER 20% INCREASE)**						
0–20%	3,144	1,281	173	5.50	13.51	2.0 (1.0–7.0)
20–40%	11,790	4,628	734	6.23	15.86	3.0 (1.0–9.0)
40–60%	3,397	1,359	197	5.80	14.50	4.0 (1.0–11.0)
**MEDIAN HOUSEHOLD INCOME (PER $20,000 INCREASE)**						
0–40,000	1,193	432	82	6.87	18.98	3.0 (1.0–8.0)
40,000–60,000	9,329	3,699	538	5.77	14.54	3.0 (1.0–8.0)
60,000–80,000	5,823	2,355	383	6.58	16.26	3.0 (1.0–9.0)
80,000–100,000	1,986	782	101	5.09	12.92	4.0 (1.0–14.0)
**SMOKING STATUS (PER 10% INCREASE)**						
0–10%	785	292	49	6.24	16.78	3.0 (1.0–7.0)
10–20%	12,201	4,829	705	5.78	14.60	3.0 (1.0–9.0)
20–30%	4,969	1,986	324	6.52	16.31	3.0 (1.0–8.0)
30–40%	376	161	26	6.91	16.15	3.0 (0.0–10.0)

*CI, confidence interval, IQR, interquartile range*.

a *Including Asian and American Indians*.

b *Including linitisplastica, hepatoid adenocarcinoma, adenosquamous carcinoma and so on*.

c *According to the eighth edition of the AJCC Cancer Staging manual*.

d *Including subtotal gastrectomy only, total gastrectomy only and radical surgery*.

e *Including divorced, separated, single (never married), and widowed*.

On univariable logistic regression (Table S2) among the entire cohort, there were nine factors that showed significance (*P* value < 0.05). They were age, gender, primary site, Lauren classification, T staging, N staging, tumor size, number of extrapulmonary metastatic sites to liver, bone, and brain and insurance situation. We put them on multivariable logistic regression which showed that age, primary site, Lauren classification, T staging, N staging, and number of extrapulmonary metastatic sites to liver, bone, and brain had significance among the entire cohort and primary site, Lauren classification, N staging, tumor size and number of extrapulmonary metastatic sites to liver, bone, and brain had significance among the subset with metastatic disease to any distant site.

On the multivariable logistic regression (Table 2) among the entire cohort, T4 (vs. T1; OR, 1.27; 95%CI, 1.02–1.57; *P* = 0.03), N1 (vs. N0; OR, 1.39; 95%CI, 1.24–1.63; *P* < 0.001), 1 extrapulmonary metastatic site (vs. 0 extrapulmonary metastatic site; OR, 4.56; 95%CI, 3.92–5.31; *P* < 0.001), 2 extrapulmonary metastatic sites (vs. 0 extrapulmonary metastatic site; OR, 13.41; 95%CI, 10.40–17.28; *P* < 0.001), 3 extrapulmonary metastatic sites (vs. 0 extrapulmonary metastatic site; OR, 21.64; 95%CI, 8.35–56.11; *P* < 0.001) were associated with significantly greater odds of having pulmonary metastases at diagnosis. And, insurance status was not associated with a risk of pulmonary metastases at diagnosis in the multivariable model. While, age 41–60 years (vs. age 18–40 years; OR, 0.70; 95%CI, 0.52–0.94; *P* = 0.02), age 61–80 years (vs. age 18–40 years; OR, 0.72; 95%CI, 0.54–0.97; *P* = 0.03) and age 80+ years (vs. age 18–40 years; OR, 0.54; 95%CI, 0.37–0.78; *P* = 0.001), middle 1/3 of stomach (vs.: upper 1/3 of stomach; OR, 0.58; 95%CI, 0.44–0.76; *P* < 0.001), lower 1/3 of stomach (vs.: upper 1/3 of stomach; OR, 0.50; 95%CI, 0.41–0.62; *P* < 0.001), and overlapping lesion (vs.: upper 1/3 of stomach; OR, 0.70; 95%CI, 0.53–0.91; *P* = 0.01), diffused-type (vs. intestinal-type; OR, 0.83; 95%CI, 0.70–0.98; *P* = 0.03), T2 (vs. T1; OR, 0.58; 95%CI, 0.40–0.84; *P* = 0.004), T3 (vs. T1; OR, 0.65; 95%CI, 0.51–0.82; *P* < 0.001), N2 (vs. N0; OR, 0.64; 95%CI, 0.45–0.92; *P* = 0.02) were associated with marginally lower odds of pulmonary metastases at diagnosis. The multivariable logistic regression of subset with metastatic disease was also showed in Table 2.

**Table 2:** Multivariable logistic regression for the presence of pulmonary metastases at diagnosis of gastric cancer.

**Variable**	**Patients, no**.		**Among entire cohort**		**Among subset with metastatic disease**	
	**Patients (*n* =18,331)**	**With pulmonary metastases (*n* = 1,104)**	**OR (95% CI)**	***P* Value**	**OR (95% CI)**	***P* Value**
**AGE AT DIAGNOSIS, YEARS**						
18–40	773	63	1 (Reference)	NA	NA	NA
41–60	6,039	385	0.70 (0.52–0.94)	0.02	NA	NA
61–80	9,682	581	0.72 (0.54–0.97)	0.03	NA	NA
80+	1,837	75	0.54 (0.37–0.78)	0.001	NA	NA
**GENDER**						
Female	6,347	331	1 (Reference)	NA	1 (Reference)	NA
Male	11,984	773	1.01 (0.87–1.16)	0.95	1.07 (0.92–1.24)	0.37
**PRIMARY SITE**						
Upper 1/3	7,027	553	1 (Reference)	NA	1 (Reference)	NA
Middle 1/3	1,676	70	0.58 (0.44–0.76)	<0.001	0.52 (0.39–0.68)	<0.001
Lower 1/3	3,808	119	0.50 (0.41–0.62)	<0.001	0.52 (0.42–0.65)	<0.001
Overlapping lesion	1,424	72	0.70 (0.53–0.91)	0.01	0.58 (0.44–0.76)	<0.001
Unknown	4,396	290	0.80 (0.68–0.95)	0.01	0.71 (0.60–0.84)	<0.001
**LAUREN CLASSIFICATION**						
Intestinal–type	11,878	802	1 (Reference)	NA	1 (Reference)	NA
Diffuse–type	5,552	232	0.83 (0.70–0.98)	0.03	0.70 (0.59–0.83)	<0.001
Others^a^	901	70	0.97 (0.76–1.31)	0.24	0.99 (0.75–1.31)	0.96
**T STAGING^b^**						
T1	4,373	222	1 (Reference)	NA	1 (Reference)	NA
T2	1,650	35	0.58 (0.40–0.84)	0.004	0.76 (0.51–1.12)	0.16
T3	4,777	128	0.65 (0.51–0.82)	<0.001	0.78 (0.61–1.00)	0.05
T4	3,672	214	1.27 (1.02–1.57)	0.03	0.96 (0.77–1.19)	0.71
Unknown	3,859	505	1.36 (1.13–1.64)	0.001	0.97 (0.81–1.17)	0.78
**N STAGING^b^**						
N0	7,775	362	1 (Reference)	NA	1 (Reference)	NA
N1	5,210	442	1.39 (1.24–1.63)	<0.001	1.12 (0.96–1.32)	0.15
N2	1,780	38	0.64 (0.45–0.92)	0.02	0.63 (0.44–0.92)	0.02
N3	1,893	49	0.77 (0.56–1.08)	0.13	0.79 (0.57–1.11)	0.18
Unknown	1,673	213	1.39 (1.13–1.69)	0.001	1.16 (0.95–1.41)	0.15
**TUMOR SIZE, CM**						
0–2	2,186	59	1 (Reference)	NA	1 (Reference)	NA
2–5	4,741	198	1.25 (0.92–1.71)	0.16	0.78 (0.56–1.09)	0.15
5+	3,742	161	1.26 (0.91–1.75)	0.16	0.73 (0.52–1.02)	0.06
Unknown	7,662	686	1.64 (1.23–2.19)	0.001	0.80 (0.59–1.09)	0.15
**EXTRAPULMONARY METASTATIC SITES TO LIVER, BONE, AND BRAIN, NO**.						
0	14,442	379	1 (Reference)	NA	1 (Reference)	NA
1	3,290	526	4.56 (3.92–5.31)	<0.001	1.22 (1.05–1.42)	0.01
2	340	128	13.41 (10.40–17.28)	<0.001	3.68 (2.86–4.74)	<0.001
3	18	10	21.64 (8.35–56.11)	<0.001	6.48 (2.51–16.69)	0.002
Unknown	241	61	8.02 (5.81–11.08)	<0.001	3.27 (2.35–4.54)	<0.001
**INSURANCE SITUATION**						
Yes	17,022	1,007	1 (Reference)	NA	NA	NA
No	922	73	1.12 (0.85–1.46)	0.42	NA	NA
Unknown	387	24	0.95 (0.61–1.48)	0.82	NA	NA

*CI, confidence interval*.

a *Including linitisplastica, hepatoid adenocarcinoma, adenosquamous carcinoma and so on*.

b *According to the eighth edition of the AJCC Cancer Staging manual*.

In order to further expound the performance of multivariable logistic regression model, binary-dependent ROC analysis was performed for the model and different variables. The model was a combination of six significant variables (age at diagnosis, Lauren classification, primary site, T staging, N staging, and extent of extrapulmonary metastastic disease) on multivariable logistic regression. Delong's test was developed to verify the performance. The value of AUC of the model (AUC: 0.775, 95%CI: 0.760–0.790) showed better than age (AUC: 0.529, 95%CI: 0.512–0.547), Lauren classification (AUC: 0.537, 95%CI: 0.520–0.554), primary site (AUC: 0.539, 95%CI: 0.521–0.557), T staging (AUC: 0.637, 95%CI: 0.619–0.656), N staging (AUC: 0.547, 95%CI: 0.530–0.565), and extent of extrapulmonary metastastic disease (AUC: 0.745, 95%CI: 0.728–0.762) in the entire cohort. All *P* value was smaller than 0.001 on Delong's test. More detail was showed in Table S4. And the ROC curves for the entire cohort and subcohort were in Figures S1, S2.

### Survival Analysis

**Figure 2:**
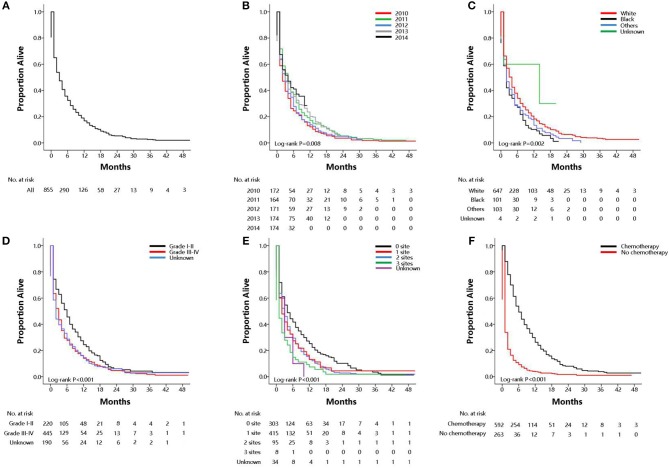
Overall survival among patients with GCPM at diagnosis (**A** overall), stratified by year at diagnosis **(B)**, race **(C)**, pathology grade **(D)**, extent of extrapulmonary metastastic disease **(E)**, chemotherapy **(F)**.

Among the subset with pulmonary metastases, there were five factors that were significantly associated with overall survival on multivariable Cox regression model. Table S3 showed univariate analysis for all-cause mortality and gastric cancer-specific mortality among GCPM. On multivariable Cox regression (Table 3) for all-cause mortality among patients with GCPM at diagnosis, black race (vs. white race; HR, 1.26; 95%CI, 1.03–1.54; *P* = 0.03), grade III-IV (vs. grade I-II; HR, 1.46; 95%CI, 1.24–1.72; *P* < 0.001), 1 extrapulmonary metastatic site (vs. 0 extrapulmonary metastatic site; HR, 1.40; 95%CI, 1.21–1.63; *P* < 0.001) and 2 extrapulmonary metastatic sites (vs. 0 extrapulmonary metastatic site; HR, 1.67; 95%CI, 1.33–2.10; *P* < 0.001), absence of chemotherapy (vs. chemotherapy; HR, 3.70; 95%CI, 3.18–4.30; *P* < 0.001) were significantly associated with an increased all-cause mortality. However, 2011 (vs. 2010; HR, 0.77; 95%CI, 0.63–0.94; *P* = 0.01), 2013 (vs. 2010; HR, 0.73; 95%CI, 0.59–0.88; *P* = 0.002), 2014 (vs. 2010; HR, 0.74; 95%CI, 0.59–0.92; *P* = 0.01) was significantly associated with a decreased all-cause mortality. And absence of surgery (vs. surgery; HR, 1.62; 95%CI, 1.13–2.33; *P* = 0.01) were significantly associated with an increased gastric cancer-specific mortality only. Gastric cancer-specific mortality among patients with GCPM at diagnosis was also presented in Table 3. Survival estimates of overall (Figure 2A) and as stratified by year at diagnosis (Figure 2B), race (Figure 2C), pathology grade (Figure 2D), extent of extrapulmonary metastastic disease (Figure 2E), and chemotherapy (Figure 2F) were graphically displayed in the Figure 2.

**Table 3:** Multivariable analysis for all-cause mortality and gastric cancer-specific mortality among patients with pulmonary metastases.

**Variable**	**Patients, no**.		**All–cause mortality**		**Gastric cancer–specific mortality**	
	**Patients (*n* = 18,331)**	**With pulmonary metastases (*n* = 1,098)**	**Hazard ratio (95% CI)**	***P* value**	**Hazard ratio (95% CI)**	***P* value**
**YEAR AT DIAGNOSIS**						
2010	3,492	221	1 (Reference)	NA	1 (Reference)	NA
2011	3,502	195	0.77 (0.63–0.94)	0.01	0.91(0.75–1.11)	0.35
2012	3,751	214	0.85 (0.70–1.04)	0.12	0.89 (0.73–1.08)	0.24
2013	3,739	229	0.73 (0.59–0.88)	0.002	0.81 (0.66–0.99)	0.04
2014	3,847	239	0.74 (0.59–0.92)	0.01	0.71 (0.57–0.90)	0.004
**RACE**						
White	12,735	816	1 (Reference)	NA	1 (Reference)	NA
Black	2,426	138	1.26 (1.03–1.54)	0.03	0.95 (0.76–1.19)	0.67
Others^a^	3,049	139	1.14 (0.93–1.39)	0.21	1.18 (0.97–1.43)	0.10
Unknown	121	5	0.77 (0.27–2.64)	0.77	0.95 (0.32–2.87)	0.93
**PRIMARY SITE**						
Upper 1/3	7,027	553	1 (Reference)	NA	1 (Reference)	NA
Middle 1/3	1,676	69	1.27(0.96–1.67)	0.10	1.43(1.15–1.78)	0.001
Lower 1/3	3,808	118	1.00 (0.80–1.24)	0.97	1.08 (0.87–1.34)	0.49
Overlapping lesion	1,424	72	1.14 (0.86–1.51)	0.35	1.37 (1.04–1.80)	0.02
Unknown	4,396	286	0.97 (0.82–1.15)	0.73	0.95 (0.79–1.14)	0.59
**PATHOLOGY GRADE**						
I–II	4,742	269	1 (Reference)	NA	1 (Reference)	NA
III–IV	10,623	572	1.46 (1.24–1.72)	<0.001	1.35 (1.15–1.58)	<0.001
Unknown	2,966	257	1.47 (1.21–1.78)	<0.001	1.33 (1.09–1.62)	0.004
**RADIOTHERAPY**						
Yes	5,416	222	1 (Reference)	NA	1 (Reference)	NA
No	12,915	876	1.09 (0.92–1.30)	0.32	1.04 (0.90–1.20)	0.62
**SURGERY^b^**						
Yes	8,453	51	NA	NA	1 (Reference)	NA
No	9,878	1,047	NA	NA	1.62 (1.13–2.33)	0.01
**CHEMOTHERAPY**						
Yes	10,495	631	1 (Reference)	NA	1 (Reference)	NA
No	7,836	467	3.32 (2.87–3.84)	<0.001	2.50 (2.16–2.91)	<0.001
**EXTRAPULMONARY METASTATIC SITES TO LIVER, BONE, AND BRAIN, NO**.						
0	14,442	377	1 (Reference)	NA	1 (Reference)	NA
1	3,290	524	1.40 (1.21–1.63)	<0.001	1.32 (1.14–1.52)	<0.001
2	340	127	1.67 (1.33–2.10)	<0.001	1.57 (1.25–1.96)	<0.001
3	18	10	1.64 (0.97–3.11)	0.13	2.03 (1.17–3.54)	0.01
Unknown	241	60	1.44 (1.07–1.93)	0.02	1.01 (0.68–1.49)	0.97
**MARITAL STATUS**						
Married	10,618	626	1 (Reference)	NA	1 (Reference)	NA
Unmarried^c^	6,789	436	0.98 (0.85–1.12)	0.76	1.01 (0.88–1.17)	0.85
Unknown	924	36	0.81 (0.55–1.18)	0.27	0.77 (0.45–1.22)	0.26
**RESIDENCE TYPE**						
Rural	467	27	NA	NA	1 (Reference)	NA
Urban	17,864	1,071	NA	NA	0.69 (0.50–0.93)	0.01
Bachelor education (per 20% increase)	18,331	1,098	NA	NA	1.02 (0.89–1.17)	0.74
Median household income (per $20,000 increase)	18,331	1,098	NA	NA	0.89 (0.80–0.99)	0.03

CI, confidence interval.

a Including Asian and American Indians.

b Including subtotal gastrectomy only, total gastrectomy only and radical surgery.

c *Including divorced, separated, single (never married), and widowed*.

## Discussion

This study analyzed the frequency and survival of gastric cancer patients with pulmonary metastases at their initial diagnosis using data from the SEER database. We also characterized the predictive factors and prognostic factors in an attempt to better understand the clinical impact of pulmonary metastases. To the best of our knowledge, this was the largest study including 1,104 patients with GCPM at present.

Previously published data had evaluated the incidence proportions and prognosis of GCPM roughly, and the frequency of pulmonary metastases from gastric cancer had yielded varying results, rang from 0.5 to 16% in current clinical practice (6, 7). However, the frequency of pulmonary metastases was found to be 22–52% at postmortem examination (9, 10). Most studies above were small samples from a single institution, which was unconvincing (6–10). Therefore a study based on population level to describe the frequency and prognosis of patients who presented with *de novo* pulmonary metastases was urgently needed. In this large retrospective study, we found that 6.02% of patients with gastric cancer had pulmonary metastases at diagnosis, and 15.19% of those with any metastases at diagnosis had pulmonary metastases. This result was a little different from that of previous published studies (6–10), but was in accordance with that of a previous study (12) using SEER database, which showed 5.92% of PM in all patients and 14.45% of PM in metastatic disease. Part of asymptomatic patients with lung metastases could not be found at initial diagnosis due to lack of accurate evaluation. On the other hand, most of the patients in previous studies developed pulmonary metastases in their disease course after a diagnosis of early-stage gastric cancer, so these researches contained both synchronous and asynchronous metastatic patients. And our work only focused on patients with metastatic gastric cancer at initial diagnosis, so the frequency of PM may be underestimated.

Risk factors for the presence of PM at GC diagnosis were determined using multivariate logistic regression. We found that patients had significantly greater odds of having pulmonary metastases at diagnosis when they showed the six factors as follow: younger, upper 1/3 of stomach, intestinal-type, T4 staging, N1 staging, and presence of more extrapulmonary metastases to liver, bone, and brain. Younger patients were always accompanied with more aggressive tumors which led to the common appearance of pulmonary metastases, as we guessed. An USA study by Smith found that 81% of young patients developed distant metastases compared to 50% in the elder for 15-year follow up which believed that earlier diagnosis and effective treatments were urgently needed to decrease the extreme lethality in these young patients (20). The presence of intestinal-type seemed to be associated with pulmonary metastases in this study. Huachuan et al. guessed that it might attribute to high expressions of extracellular matrix metalloproteinase inducer (EMMPRIN), which promoted tumor growth and metastasis (21). Primary tumor located at the upper 1/3 of stomach had significantly higher percentage of pulmonary metastases could be attributed to “seed-and-soil” hypothesis (“seed-and-soil” hypothesis implies organ specific tropism of circulating tumor cells) (22). Patients with T4 staging and N1 staging were easier to diagnose with pulmonary metastases, too. The finding was only seen in N1 staging because of lack of patients with N2 staging (*N* = 37) and N3 staging (*N* = 45) we guessed. And most N staging of this study was based on clinical staging which may not be accurate enough (23–25). Moreover, only T4 staging had a higher proportion of lung metastases compared with T1 staging. We thought that the same reasons existed in the variable of T staging. As we know, TNM staging was visibly associated with survival in GC. Thus, we inferred that later T staging and N staging may be associated with poor prognosis in GCPM. However, these results should be confirmed with further studies carefully. Besides, patients presented with more extrapulmonary metastatic sites were associated with a higher proportion of lung metastases. A similar result was also indicated in breast cancer (26). To say the least, our study indicated that GC patients with high risk factors above need further examination at first diagnosis, like chest CT, or PET-CT. However, it was unclear whether early detection could contribute to a more favorable survival significantly.

The multivariate logistic regression model including six significant variables had the best predictive value, with an AUC value of 0.775. And the AUC value of single predictors ranged from 0.529 to 0.745. From them, a large extent of extrapulmonary metastases hold a maximum AUC value of 0.745, and age had a minimum AUC value of 0.529. These predictors with AUC smaller than 0.6 were best to further evaluate. However, the model contains six significant variables that had an acceptable performance to predict the presence of PM in our study, which had not been reported yet.

Prognostic factors of PM at GC diagnosis were analyzed using the multivariate Cox model. We found that patients had a significantly higher risk of mortality when they showed the five factors as follows: diagnosis at previous period, black race, adverse pathology grade, absence of chemotherapy and presence of more extrapulmonary metastases to liver, bone and brain. The prognosis was better for those patients diagnosed at a later period, which may owe to those patients who receive more effective treatment with the improvement of medical conditions in recent years (2). It was worth noting that black patients had worse overall median survival which may be related to genetics and economic conditions which had not been well-explained in previous literature. And GCPM patients with adverse pathological grade and more metastatic sites predicted significantly poor survival in this study. This result had not been well-reported by published studies to the best of our knowledge. The median OS was 3.0 months from initial diagnosis of GCPM in the SEER, which was similar to the previous study (12). Chemotherapy was considered the basic treatment for advanced gastric cancer at present. The median OS of patients with and without chemotherapy was 6 and 1 months, separately, in this study. We can find a significant increase in the hazard ratio for all-cause mortality (2.87–3.84; *P* < 0.001) and gastric cancer-specific mortality (2.16–2.91; *P* < 0.001) among absence of chemotherapy vs. presence of chemotherapy. However, the role of surgery in GCPM had not been effectively identified yet. Only a few studies and case reports (8, 10, 11, 16, 17) proposed that radical surgery may improve quality of life and survival in highly selected cases with isolated pulmonary metastases, while others hold a different sound (27, 28). And our study found that surgery showed significant benefit in gastric cancer-specific mortality analysis only. The hazard ratio (1.13–2.33; *P* = 0.01) had a significant increase from absence of surgery to presence of surgery on a competitive risk model, while showed no significance on all-cause mortality analysis. What's more, the median OS had no significant increase from absence of surgery group (3 months) to surgery group (4 months), which may have had four reasons as follows. Firstly, most patients in published studies were confirmed pulmonary metastases after a diagnosis of early-stage gastric cancer and received metastasectomy later (6–10). Secondly, those patients in published studies were highly selected with excellent surgical conditions. Thirdly, samples in previous reports were really small with 12 patients as the largest sample (8). Finally, the GCPM patients with surgical resection were only 51 in this study, among them forty-four patients received gastrectomy and only 7 patients received radical surgery whose median survival was 6.0months (IQR:1-27mo), which needs further investigation with more patients and convincing research methods. A prospective randomized controlled trial (RCT) was not easy to conduct for patients with GCPM due to their complex characteristics, so the road may be hard and long. Besides, radiotherapy showed no significance for overall survival on multivariate Cox model in this study. In summary, chemotherapy may be the basic treatment for GCPM at present, while surgery may be available for those highly selected patients with caution. And we did not recommend routine surgery and radiotherapy at present.

Although our study was based on population-level, containing a large number of cases, we should not ignore its limitations.

Firstly, this study was a retrospective study. We could know patients with metastatic disease to bone, liver, lung and brain, but the SEER database did not provide information about other metastatic sites, like peritoneal metastases. Moreover, we only had information on synchronous metastasis to lung, lack of a relative minority compared to those patients who may develop asynchronous metastases. Secondly, information relating to comorbidities, performance status was not available in the SEER database. Thirdly, residence type, education level, and median household income were defined at a county level, not a patient level, possibly affecting the results of the logistic and Cox regressions. Fourthly, more detail information about radiotherapy, surgery and chemotherapy were not reported in the SEER database. Finally, the SEER did not record the details of pulmonary metastases.

To the best of our knowledge, this study was the first population-based analysis of patients with pulmonary metastases at initial diagnosis of gastric cancer. It provided important suggestions for clinicians to consider designing studies that evaluate the utility of screening among patients with higher risk of pulmonary metastases. The prognostic factors on GCPM were analyzed in this study too. Besides, we described the significance of different treatment on GCPM, which might provide some help to clinical practice.

## Conclusions

In summary, the findings of this study based on a population level provided estimates of the frequency for GCPM at time of diagnosis. Patients present with younger, upper 1/3 of stomach, intestinal-type, T4 staging, N1 staging, and presence of more extrapulmonary metastases to liver, bone, and brain had significantly greater odds of having pulmonary metastases at diagnosis. A series of risk factors for PM in GC patients were identified, which can indicate routine screening in such patients. Furthermore, a list of prognostic factors for GCPM patients by survival estimates was found. GCPM patients present with black race, diagnosis at previous period, adverse pathology grade, presence with more extrapulmonary metastases to liver, bone and brain and absence of chemotherapy had a significantly higher risk of mortality. These finding can signify the need for individualized treatment for these patients. Chemotherapy may be the basic treatment for GCPM at present, while surgery may be available for those highly selected patients with caution. And we do not recommend routine surgery and radiotherapy at present.

## Data Availability

Publicly available datasets were analyzed in this study. This data can be found here: https://seer.cancer.gov/data/.

## Ethics Statement

The SEER was public-use data: informed consent was waived. And our study was deemed exempt from institutional review board approval by NanFang Hospital, Southern Medical University.

## Author Contributions

All authors listed had made a substantial contribution to the work. YJ and GL put forward the conception and designed the study. WH, TL, and ZH collected and collated the data. ZS, HL, and JY analyzed data and wrote the manuscript together. YH, HC, and MZ made contribution to proofread the article. Finally, all the authors take responsible to the final manuscript and approved it for publication.

### Conflict of Interest Statement

The authors declare that the research was conducted in the absence of any commercial or financial relationships that could be construed as a potential conflict of interest.
