# Supplementary material for: Frequency and Prognosis of Pulmonary Metastases in Newly Diagnosed Gastric Cancer
Source: Front Oncol. 2019 Jul 30;9:671. doi: 10.3389/fonc.2019.00671 (PMC6683847; doi:10.3389/fonc.2019.00671)

Supplementary Materials

I. Supplementary tables: 4

II. Supplementary figures: 2

Table S1. Chi-square or Fisher's Test for Clinical Characteristics of Gastric Cancer Patients with or without PM.

Variables	Entire cohort (with gastric cancer), n=18331				Subcohort (with metastatic disease to any distant site), n=7268			
	Without PM	With PM	Total	P Value[f]	Without PM	With PM	Total	P Value[f]
**Year at diagnosis**				**0.624**				**0.876**
2010	3271 (18.99%)	221 (20.02%)	3492		1188 (19.27%)	221 (20.02%)	1409	
2011	3307 (19.20%)	195 (17.66%)	3502		1157 (18.77%)	195 (17.66%)	1352	
2012	3533 (20.51%)	218 (19.75%)	3751		1251 (20.30%)	218 (19.75%)	1469	
2013	3510 (20.37%)	229 (20.74%)	3739		1247 (20.23%)	229 (20.74%)	1476	
2014	3606 (20.93%)	241 (21.83%)	3847		1321 (21.43%)	241 (21.83%)	1562	
**Age at diagnosis, Y**				**0.000**				**0.022**
18-40	710 (4.12%)	63(5.71%)	773		388 (6.29%)	63 (5.71%)	451	
41-60	5654 (32.82%)	385 (34.87%)	6039		2378 (38.58%)	385 (34.87%)	2763	
61-80	9101 (52.83%)	581 (52.62%)	9682		2931 (47.55%)	581 (52.63%)	3512	
80+	1762 (10.23%)	75 (6.79%)	1837		467 (7.58%)	75 (6.79%)	542	
**Race**				**0.000**				**0.057**
White	11915 (69.63%)	820 (74.61%)	12735		4362 (71.14%)	820 (74.61%)	5182	
Black	2287 (13.37%)	139 (12.65%)	2426		858 (13.99%)	139 (12.65%)	997	
Others[a]	2909 (17.00%)	140 (12.74%)	3049		912 (14.87%)	140 (12.74%)	1052	
Unknown	116	5	121		32	5	37	
**Original**				**0.207**				**0.002**
Hispanic	3646 (21.16%)	216 (19.57%)	3862		1474 (23.91%)	216 (24.86%)	1690	
Non-Hispanic	13581 (78.84%)	888 (80.43%)	14469		4690 (76.09%)	888(75.14%)	5578	
**Gender**				**0.001**				**0.000**
Male	11211 (65.08%)	773 (70.02%)	11984		3970 (66.84%)	773 (67.28%)	4743	
Female	6016 (34.92%)	331 (29.98%)	6347		2194 (33.16%)	331 (32.72%)	2525	
**Primary site**				**0.000**				**0.000**
Upper 1/3	6474 (49.34%)	553 (67.94%)	7027		2128 (48.13%)	553 (67.94%)	2681	
Middle 1/3	1606 (12.24%)	70 (8.60%)	1676		624 (14.11%)	70 (8.60%)	694	
Lower 1/3	3689 (28.12%)	119 (14.62%)	3808		1067 (24.13%)	119 (14.62%)	1186	
Overlapping lesion	1352 (10.30%)	72 (8.85%)	1424		602 (13.62%)	72 (8.85%)	674	
Unknown	4106	290	4396		1743	290	2033	
**Pathology grade**				**0.529**				**0.000**
I-II	4473 (30.81%)	269 (31.83%)	4742		1128 (23.46%)	269 (31.83%)	1397	
III-IV	10047 (69.19%)	576 (68.17%)	10623		3680 (76.54%)	576 (68.17%)	4256	
Unknown	2707	259	2966		1356	259	1615	
**Lauren classification**				**0.000**				**0.000**
Intestinal-type	11076 (64.29%)	802 (72.64%)	11878		3819 (85.20%)	802 (72.37%)	4621	
Diffuse-type	5320 (30.88%)	232 (21.01%)	5552		2009 (12.24%)	232 (22.59%)	2241	

Others[b]	831 (4.82%)	70 (6.34%)	901		336 (5.45%)	70 (6.34%)	406	
**Tumor staging[c]**				**NA**				**NA**
I	3315 (20.48%)	0	3315		0	0	0	
II	2359 (14.57%)	0	2359		0	0	0	
III	4351 (26.88%)	0	4351		0	0	0	
IV	6164 (38.08%)	1104	7268		6164	1104	7268	
Unknown	1038	0	1038		0	0	0	
**T staging**				**0.000**				**0.000**
T1	4151 (29.92%)	222 (37.06%)	4373		1080 (28.88%)	222 (37.06%)	1302	
T2	1615 (11.64%)	35 (5.84%)	1650		298 (7.97%)	35 (5.84%)	333	
T3	4649 (33.51%)	128 (21.39%)	4777		934 (24.97%)	128 (21.39%)	1062	
T4	3458 (24.93%)	214 (35.73%)	3672		1428 (38.18%)	214 (35.73%)	1642	
Unknown	3354	505	3859		2424	505	2929	
**N staging**				**0.000**				**0.000**
N0	7413 (47.02%)	362 (40.63%)	7775		2186 (42.51%)	362 (40.63%)	2548	
N1	4768 (30.24%)	442 (49.61%)	5210		2130 (41.42%)	442 (49.61%)	2572	
N2	1742 (11.05%)	38 (4.26%)	1780		379 (7.37%)	38 (4.26%)	417	
N3	1844 (11.70%)	49 (5.50%)	1893		447 (8.69%)	49 (5.50%)	496	
Unknown	1460	213	1673		1022	213	1235	
**M staging**				**NA**				**NA**
M0	11063 (64.22%)	0	11063		0	0	0	
M1	6164 (35.78%)	1104 (100%)	7268		6164 (100%)	1104 (100%)	7268	
**Surgery[d]**				**0.000**				**0.000**
Yes	8402 (48.77%)	51 (4.62%)	8453		832 (13.50%)	51 (4.62%)	883	
No	8825 (51.23%)	1053 (95.38%)	9878		5332 (86.50%)	1053 (95.38%)	6385	
**Radiotherapy**				**0.000**				**0.006**
Yes	5194 (30.15%)	222 (20.11%)	5416		1029 (16.69%)	222 (20.11%)	1251	
No	12033 (69.85%)	882 (79.89%)	12915		5135 (83.31%)	882 (79.89%)	6017	
**Chemotherapy**				**0.946**				**0.016**
Yes	9864 (57.26%)	631 (57.16%)	10495		3760 (61.00%)	631 (57.16%)	4391	
No	7363 (42.74%)	473 (42.84%)	7836		2404 (39.00%)	473 (42.84%)	2877	
**Tumor size, cm**				**0.004**				**0.042**
0-2	2127 (20.75%)	59 (14.11%)	2186		263 (10.92%)	59 (14.11%)	322	
2-5	4543 (44.32%)	198 (47.37%)	4741		1080 (44.85%)	198 (47.37%)	1278	
5+	3581 (34.93%)	161 (38.52%)	3742		1065 (44.23%)	161 (38.52%)	1226	
Unknown	6976	686	7662		3756	686	4442	
**Extrapulmonary metastatic sites to liver, bone, and brain, No.**				**0.000**				**0.000**
0	14063 (82.50%)	379 (36.34%)	14442		3043 (50.49%)	379 (36.34%)	3422	
1	2764 (16.21%)	526 (50.43%)	3290		2764 (45.86%)	526 (50.43%)	3290	
2	212 (1.24%)	128 (12.27%)	340		212 (3.52%)	128 (12.27%)	340	
3	8 (0.05%)	10 (0.96%)	18		8 (0.13%)	10 (0.96%)	18	

Unknown	180	61	241		137	61	198	
**Marital status**				**0.113**				**0.252**
Married	9991 (61.15%)	627 (58.71%)	10618		3567 (60.57%)	627 (58.71%)	4194	
Unmarried[e]	6348 (38.85%)	441 (41.29%)	6789		2322(39.43%)	441 (41.29%)	2763	
Unknown	888	36	924		275	36	311	
**Insurance situation**				**0.045**				**0.289**
Yes	16015 (94.96%)	1007 (93.24%)	17022		5654 (91.76%)	1007 (93.24%)	6661	
No	849 (5.04%)	73 (6.76%)	922		416 (6.75%)	73 (6.76%)	489	
Unknown	363	24	387		2	24	118	
**Residence type**				**0.825**				**0.659**
Rural	440 (2.55%)	27 (2.45%)	467		165 (2.68%)	27 (2.45%)	192	
Urban	16787 (97.45%)	1077 (97.55%)	17864		5999 (97.32%)	1077 (97.55%)	7076	
**Bachelor education (per 20% increase)**				**0.264**				**0.084**
0-20%	2971 (17.25%)	173 (15.67%)	3144		1108 (17.98%)	173 (15.67%)	1281	
20-40%	11056 (64.18%)	734 (66.49%)	11790		3894 (63.17%)	734 (66.49%)	4628	
40-60%	3200 (18.58%)	197 (17.84%)	3397		1162 (18.85%)	197 (17.84%)	1359	
**Median household income (per $20,000 increase)**				**0.031**				**0.010**
0-40,000	1111 (6.45%)	82 (7.43%)	1193		350 (5.68%)	82 (7.43%)	432	
40,000-60,000	8791 (51.03%)	538 (48.73%)	9329		3161 (51.28%)	538 (48.73%)	3699	
60,000-80,000	5440 (31.58%)	383 (34.69%)	5823		1972 (31.99%)	383 (34.69%)	2355	
80,000-100,000	1885 (10.94%)	101 (9.15%)	1986		681 (11.05%)	101 (9.15%)	782	
**Smoking status (per 10% increase)**				**0.255**				**0.267**
0-10%	736 (4.27%)	49 (4.44%)	785		243 (3.94%)	49 (4.44%)	292	
10-20%	11496 (6.67%)	705 (63.86%)	12201		4124 (66.90%)	705 (63.86%)	4829	
20-30%	4645 (26.96%)	324 (29.35%)	4969		1662 (26.96%)	324 (29.35%)	1986	
30-40%	350 (2.03%)	26 (2.36%)	376		135 (2.19%)	26 (2.36%)	161	

Abbreviations:

CI: confidence interval, IQR: interquartile range;

[a] including Asian and American Indians;

[b] including linitisplastica, hepatoid adenocarcinoma, adenosquamous carcinoma and so on;

[c] according to the eighth edition of the AJCC Cancer Staging manual;

[d] including subtotal gastrectomy only, total gastrectomy only and radical surgery;

[e] including divorced, separated, single (never married), and widowed;

[f] Chi-square or Fisher's Test was played at the exclusion of unknown patients.

Table S2. Univariate Logistic Regression for the Presence of Pulmonary Metastases at Diagnosis of Gastric Cancer.

Variable	Patients, No.		Among Entire Cohort		Among Subset With Metastatic Disease	
	Patients (n =18331)	With Pulmonary Metastases (n = 1104)	OR (95% CI)	P Value	OR (95% CI)	P Value
**Year at diagnosis**						
2010	3492	221	1 (Reference)	NA	1 (Reference)	NA
2011	3502	195	0.87 (0.72-1.06)	0.18	0.91 (0.74-1.12)	0.35
2012	3751	218	0.91 (0.75-1.11)	0.36	0.94 (0.76-1.15)	0.53
2013	3739	229	0.97 (0.80-1.17)	0.72	0.99 (0.81-1.21)	0.90
2014	3847	241	0.99 (0.82-1.19)	0.91	0.98 (0.80-1.20)	0.85
**Age at diagnosis, Y**						
18-40	773	63	1 (Reference)	NA	1 (Reference)	NA
41-60	6039	385	0.77 (0.58-1.01)	0.06	1.00 (0.75-1.33)	0.98
61-80	9682	581	0.72 (0.55-0.94)	0.02	1.22 (0.92-1.62)	0.16
80+	1837	75	0.48 (0.34-0.68)	<0.001	0.99 (0.69-1.42)	0.95
**Race**						
White	12735	820	1 (Reference)	NA	1 (Reference)	NA
Black	2426	139	0.88 (0.73-1.06)	0.19	0.86 (0.71-1.05)	0.13
Others[a]	3049	140	0.70 (0.58-0.84)	<0.001	0.82 (0.67-0.99)	0.04
Unknown	121	5	0.63 (0.26-1.54)	0.31	0.83 (0.32-2.14)	0.70
**Gender**						
Female	6347	331	1 (Reference)	NA	1 (Reference)	NA
Male	11984	773	1.25 (1.10-1.43)	0.001	1.29 (1.12-1.48)	<0.001
**Original**						
Hispanic	3862	216	1 (Reference)	NA	1 (Reference)	NA
Non-Hispanic	14469	888	1.10 (0.95-1.29)	0.21	1.29 (1.10-1.52)	0.002
**Primary site**						
Upper 1/3	7027	553	1 (Reference)	NA	1 (Reference)	NA
Middle 1/3	1676	70	0.51(0.40-0.66)	<0.001	0.43 (0.33-0.56)	<0.001
Lower 1/3	3808	119	0.38 (0.31-0.46)	<0.001	0.43 (0.35-0.53)	<0.001
Overlapping lesion	1424	72	0.62 (0.48-0.82)	<0.001	0.46 (0.35-0.60)	<0.001
Unknown	4396	290	0.82 (0.71-0.96)	0.01	0.64 (0.55-0.75)	<0.001
**Pathology grade**						
I-II	4742	269	1 (Reference)	NA	1(Reference)	NA
III-IV	10623	576	0.95 (0.82-1.11)	0.53	0.66 (0.56-0.77)	<0.001
Unknown	2966	259	1.59 (1.33-1.90)	<0.001	0.80 (0.66-0.97)	0.02
**Lauren classification**						

Intestinal-type	11878	802	1 (Reference)	NA	1 (Reference)	NA
Diffuse-type	5552	232	0.60 (0.52-0.70)	<0.001	0.55 (0.47-0.64)	<0.001
Others[b]	901	70	1.16 (0.90-1.50)	0.24	0.99 (0.76-1.30)	0.95
**T staging[c]**						
T1	4373	222	1 (Reference)	NA	1 (Reference)	NA
T2	1650	35	0.41 (0.28-0.58)	<0.001	0.57 (0.39-0.84)	0.004
T3	4777	128	0.52 (0.41-0.64)	<0.001	0.67 (0.53-0.84)	0.001
T4	3672	214	1.16 (0.95-1.40)	0.14	0.73 (0.60-0.89)	0.002
Unknown	3859	505	2.82 (2.39-3.32)	<0.001	1.01 (0.85-1.21)	0.88
**N staging[c]**						
N0	7775	362	1 (Reference)	NA	1 (Reference)	NA
N1	5210	442	1.90 (1.64-2.19)	<0.001	1.25 (1.08-1.46)	0.003
N2	1780	38	0.45 (0.32-0.63)	<0.001	0.61 (0.43-0.86)	0.005
N3	1893	49	0.54 (0.40-0.74)	<0.001	0.66 (0.48-0.91)	0.01
Unknown	1673	213	2.99 (2.50-3.57)	<0.001	1.26 (1.05-1.51)	0.02
**Tumor size, cm**						
0-2	2186	59	1 (Reference)	NA	1 (Reference)	NA
2-5	4741	198	1.57 (1.17-2.11)	0.003	0.82 (0.59-1.13)	0.22
5+	3742	161	1.62 (1.20-2.20)	0.002	0.67 (0.49-0.94)	0.02
Unknown	7662	686	3.55 (2.71-4.65)	<0.001	0.81 (0.61-1.09)	0.17
**Extrapulmonary metastatic sites to liver, bone, and brain, No.**						
0	14442	379	1 (Reference)	NA	1 (Reference)	NA
1	3290	526	7.06 (6.15-8.11)	<0.001	1.53 (1.33-1.76)	<0.001
2	340	128	22.40 (17.59-28.54)	<0.001	4.85 (3.80-6.19)	<0.001
3	18	10	46.38 (18.20-118.18)	<0.001	10.04 (3.94-25.59)	0.002
Unknown	241	61	12.58 (9.24-17.11)	<0.001	3.58 (2.60-4.92)	<0.001
**Insurance situation**						
Yes	17000	1007	1 (Reference)	NA	1 (Reference)	NA
No	922	73	1.37 (1.07-1.75)	0.01	0.99 (0.76-1.28)	0.91
Unknown	387	24	1.05 (0.69-1.60)	0.81	1.43 (0.91-2.26)	0.12
**Marital status**						
Married	10618	627	1(Reference)	NA	1 (Reference)	NA
Unmarried[d]	6789	441	1.11 (0.98-1.26)	0.11	1.08 (0.95-1.23)	0.25
Unknown	924	36	0.65 (0.46-0.91)	0.01	0.75 (0.52-1.07)	0.11
**Residence type**						
Rural	467	27	1 (Reference)	NA	1 (Reference)	NA
Urban	17864	1077	1.05 (0.71-1.55)	0.83	1.10 (0.73-1.66)	0.66
**Bachelor education (per 20% increase）**	18331	1104	1.02 (0.93-1.13)	0.65	1.04 (0.93-1.15)	0.51
**Median household income (per $20,000 increase)**	18331	1104	0.98 (0.90-1.06)	0.55	0.95 (0.88-1.04)	0.25

**Smoking status (per 10% increase）**	18331	1104	1.09 (0.98-1.21)	0.11	1.07 (0.96-1.20)	0.23

Abbreviations:
CI: confidence interval;

[a] including Asian and American Indians;

[b] including linitis plastica, hepatoid adenocarcinoma, adenosquamous carcinoma and so on;

[c] according to the eighth edition of the AJCC Cancer Staging manual;

[d] including divorced, separated, single (never married), and widowed.

Table S3. Univariate Analysis for All-Cause Mortality and Gastric Cancer-Specific Mortality Among Patients With Pulmonary Metastases.

Variable	Patients, No.		All-Cause Mortality		Gastric Cancer-Specific Mortality	
	Patients (n = 18331)	With Pulmonary Metastases (n = 1098)	Hazard Ratio (95% CI)	P Value	Hazard Ratio (95% CI)	P Value
**Year at diagnosis**						
2010	3492	221	1 (Reference)	NA	1 (Reference)	NA
2011	3502	195	0.80 (0.66-0.97)	0.03	0.89 (0.74-1.06)	0.17
2012	3751	214	0.92 (0.76-1.11)	0.39	0.92 (0.77-1.11)	0.39
2013	3739	229	0.80 (0.66-0.97)	0.03	0.84 (0.70-1.02)	0.07
2014	3847	239	0.72 (0.58-0.91)	0.01	0.72 (0.57-0.89)	0.003
**Age at diagnosis, Y**						
18-40	773	60	1 (Reference)	NA	1 (Reference)	NA
41-60	6039	382	0.87 (0.65-1.17)	0.35	0.93 (0.70-1.22)	0.58
61-80	9682	581	0.94 (0.70-1.25)	0.65	0.93 (0.71-1.22)	0.62
80+	1837	75	1.10 (0.77-1.58)	0.60	1.13 (0.81-1.59)	0.48
**Race**						
White	12735	816	1 (Reference)	NA	1 (Reference)	NA
Black	2426	138	1.35 (1.11-1.64)	0.002	1.17 (0.97-1.42)	0.10
Others[a]	3049	139	1.20 (0.99-1.46)	0.07	1.18 (0.99-1.42)	0.07
Unknown	121	5	0.60 (0.19-1.87)	0.38	0.70 (0.22-2.25)	0.55
**Gender**						
Male	11984	770	1 (Reference)	NA	1 (Reference)	NA
Female	6347	328	1.03 (0.89-1.18)	0.71	1.05 (0.92-1.19)	0.48
**Original**						
Hispanic	3862	213	1 (Reference)	NA	1 (Reference)	NA
Non-Hispanic	14469	885	1.07 (0.90-1.26)	0.46	1.12 (0.95-1.32)	0.18
**Primary site**						

Upper 1/3	7027	553	1 (Reference)	NA	1 (Reference)	NA
Middle 1/3	1676	69	1.32 (1.00-1.73)	0.04	1.36 (1.10-1.70)	0.01
Lower 1/3	3808	118	1.28 (1.04-1.59)	0.02	1.21 (1.00-1.46)	0.05
Overlapping lesion	1424	72	1.37 (1.05-1.79)	0.02	1.30 (0.97-1.76)	0.08
Unknown	4396	286	1.27 (1.08-1.48)	0.003	1.15 (0.98-1.35)	0.08
**Pathology grade**						
I-II	4742	269	1 (Reference)	NA	1 (Reference)	NA
III-IV	10623	572	1.38 (1.18-1.62)	<0.001	1.29 (1.12-1.49)	<0.001
Unknown	2966	257	1.42 (1.18-1.72)	<0.001	1.28 (1.07-1.53)	0.01
**Lauren classification**						
Intestinal-type	11878	801	1 (Reference)	NA	1 (Reference)	NA
Diffuse-type	5552	229	1.06 (0.90-1.25)	0.46	0.99 (0.85-1.16)	0.94
Others[b]	901	68	1.29 (0.99-1.68)	0.06	1.31 (1.03-1.66)	0.03
**T staging[c]**						
T1	4373	221	1 (Reference)	NA	1 (Reference)	NA
T2	1650	34	0.85 (0.58-1.26)	0.43	0.97 (0.65-1.46)	0.90
T3	4777	128	0.78 (0.61-0.99)	0.04	0.79 (0.64-0.98)	0.03
T4	3672	212	1.07 (0.87-1.31)	0.52	1.07 (0.89-1.29)	0.49
Unknown	3859	503	1.07 (0.90-1.27)	0.43	1.03 (0.88-1.21)	0.72
**N staging[c]**						
N0	7775	362	1 (Reference)	NA	1 (Reference)	NA
N1	5210	436	0.95 (0.82-1.11)	0.54	1.02 (0.88-1.17)	0.84
N2	1780	38	0.95 (0.65-1.38)	0.78	0.83 (0.57-1.22)	0.35
N3	1893	49	1.01 (0.72-1.40)	0.97	0.96 (0.72-1.30)	0.80
Unknown	1673	213	1.27 (1.06-1.52)	0.01	1.28 (1.08-1.51)	0.004
**Surgery[d]**						
Yes	8453	51	1 (Reference)	NA	1 (Reference)	NA
No	9878	1047	1.36 (0.98-1.88)	0.07	1.60 (1.13-2.25)	0.01
**Radiotherapy**						
Yes	5416	222	1 (Reference)	NA	1 (Reference)	NA
No	12915	876	1.32 (1.12-1.55)	0.001	1.24 (1.08-1.42)	0.002
**Chemotherapy**						
Yes	10495	631	1 (Reference)	NA	1 (Reference)	NA
No	7836	467	3.20 (2.79-3.67)	<0.001	2.40 (2.08-2.78)	<0.001
**Tumor size, cm**						
0-2	2186	59	1 (Reference)	NA	1 (Reference)	NA
2-5	4741	196	0.80 (0.58-1.12)	0.19	0.78 (0.55-1.11)	0.16
5+	3742	161	1.09 (0.78-1.52)	0.62	1.06 (0.95-1.50)	0.77
Unknown	7662	682	1.10 (0.81-1.46)	0.55	1.00 (1.00-1.39)	1.00
**Extrapulmonary metastatic sites to liver, bone, and brain, No.**						
0	14442	377	1 (Reference)	NA	1 (Reference)	NA

1	3290	524	1.35 (1.16-1.56)	<0.001	1.32 (1.15-1.51)	<0.001
2	340	127	1.37 (1.10-1.72)	0.01	1.40 (1.12-1.73)	<0.001
3	18	10	1.86 (0.99-3.49)	0.06	2.00 (1.39-2.87)	<0.001
Unknown	241	60	1.91 (1.43-2.54)	<0.001	1.34 (0.95-1.89)	0.10
**Marital status**						
Married	10618	626	1 (Reference)	NA	1 (Reference)	NA
Unmarried[e]	6789	436	1.20 (1.05-1.37)	0.01	1.17 (1.03-1.33)	0.01
Unknown	924	36	0.94 (0.65-1.37)	0.75	0.91 (0.62-1.33)	0.63
**Residence type**						
Rural	467	27	1 (Reference)	NA	1 (Reference)	NA
Urban	17864	1071	0.74 (0.50-1.10)	0.14	0.66 (0.52-0.85)	0.001
**Insurance situation**						
Yes	17000	1003	1 (Reference)	NA	1 (Reference)	NA
No	922	73	1.00 (0.76-1.31)	0.97	0.88 (0.66-1.18)	0.41
Unknown	387	22	0.97 (0.61-1.55)	0.90	0.96 (0.57-1.61)	0.88
**Bachelor education (per 20% increase)**	18331	1098	0.89 (0.79-0.99)	0.04	0.88 (0.79-0.98)	0.02
**Median household income (per $20,000 increase)**	18331	1098	0.90 (0.83-0.98)	0.02	0.88 (0.82-0.96)	0.002
**Smoking status (per 10% increase)**	18331	1098	1.02 (0.91-1.14)	0.74	1.07 (0.96-1.18)	0.24

Abbreviations:

CI: confidence interval, IQR: interquartile range;

[a] including Asian and American Indians;

[b] including linitis plastica, hepatoid adenocarcinoma, adenosquamous carcinoma and so on;

[c] according to the eighth edition of the AJCC Cancer Staging manual;

[d] including subtotal gastrectomy only, total gastrectomy only and radical surgery;

[e] including divorced, separated, single (never married), and widowed.

Table S4. The Value of ROC in the Entire Cohort and Subcohort.

Variables	Entire cohort, n=18331		Subcohort, n=7268	
	AUC (95% CI)	P Value[b]	AUC (95% CI)	P Value[b]
**Multivariable Logistic Regression model[a]**	0.775 (0.760-0.790)	NA[C]	0.628 (0.611-0.646)	NA[C]
**Extrapulmonary metastases**	0.745 (0.728-0.762)	<0.001	0.601 (0.583-0.620)	<0.001
**T staging**	0.637 (0.619-0.656)	<0.001	0.521 (0.502-0.540)	<0.001
**N staging**	0.547 (0.530-0.565)	<0.001	0.507 (0.488-0.525)	<0.001
**Primary site**	0.539 (0.521-0.557)	<0.001	0.562 (0.543-0.581)	<0.001
**Lauren classification**	0.537 (0.520-0.554)	<0.001	0.549 (0.531-0.567)	<0.001
**Age at diagnosis**	0.529 (0.512-0.547)	<0.001	0.518 (0.499-0.536)	<0.001

Abbreviations:

CI: confidence interval,

[a] a combination of six significant variables (age at diagnosis, Lauren classification, primary site, T staging, N staging and extent of extrapulmonary metastastic disease);

[b] using Delong's test;

[c] As a control group.

Figure S1: ROC curves to predict the presence of PM in the entire cohort stratified by model (AUC: 0.775), extent of extrapulmonary metastastic disease (AUC: 0.745), T staging (AUC: 0.637), N staging (AUC: 0.547), primary site (AUC: 0.539), Lauren classification (AUC: 0.537) and age at diagnosis (AUC: 0.529).

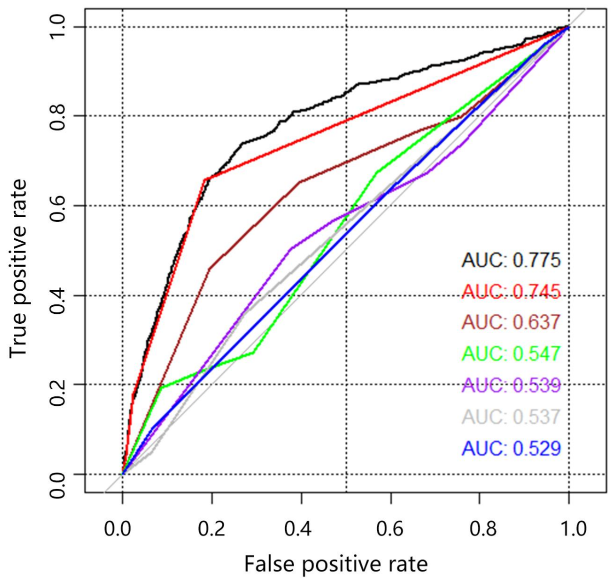

Figure S2: ROC curves to predict the presence of PM in the subcohort (with metastatic disease to any distant site) stratified by model (AUC: 0.628), extent of extrapulmonary metastastic disease (AUC: 0.601), T staging (AUC: 0.521), N staging (AUC: 0.507), primary site (AUC: 0.562), Lauren classification (AUC: 0.549) and age at diagnosis (AUC: 0.518).

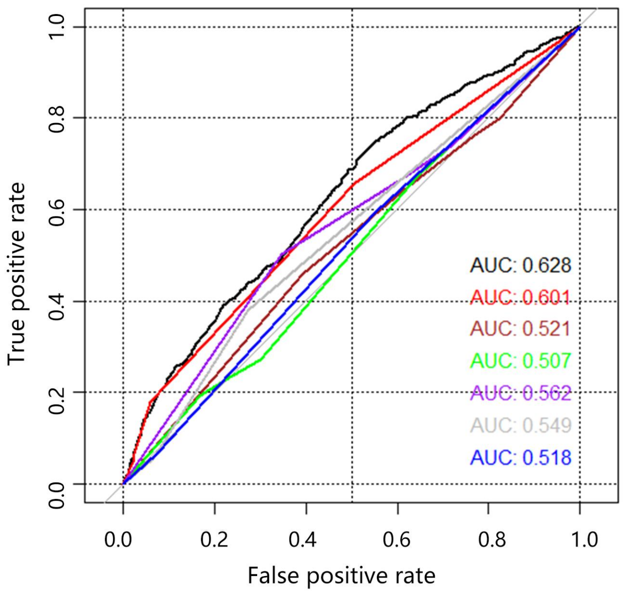

Supplement: Supplementary file 1 [file Presentation_1.pdf]
